# Versatile micro-electrode array to monitor human iPSC derived 3D neural tissues at air-liquid interface

**DOI:** 10.3389/fncel.2024.1389580

**Published:** 2024-05-09

**Authors:** Luc Stoppini, Marc O. Heuschkel, Céline Loussert-Fonta, Loris Gomez Baisac, Adrien Roux

**Affiliations:** Tissue Engineering Laboratory, HEPIA HES-SO University of Applied Sciences and Arts Western Switzerland, Geneva, Switzerland

**Keywords:** brain organoids, 3D neural tissue, electrophysiology, micro-electrode array, electrical neural activity, air-liquid interface, *in vitro* model

## Abstract

Engineered 3D neural tissues made of neurons and glial cells derived from human induced pluripotent stem cells (hiPSC) are among the most promising tools in drug discovery and neurotoxicology. They represent a cheaper, faster, and more ethical alternative to *in vivo* animal testing that will likely close the gap between *in vitro* animal models and human clinical trials. Micro-Electrode Array (MEA) technology is known to provide an assessment of compound effects on neural 2D cell cultures and acute tissue preparations by real-time, non-invasive, and long-lasting electrophysiological monitoring of spontaneous and evoked neuronal activity. Nevertheless, the use of engineered 3D neural tissues in combination with MEA biochips still involves series of constraints, such as drastically limited diffusion of oxygen and nutrients within tissues mainly due to the lack of vascularization. Therefore, 3D neural tissues are extremely sensitive to experimental conditions and require an adequately designed interface that provides optimal tissue survival conditions. A well-suited technique to overcome this issue is the combination of the Air-Liquid Interface (ALI) tissue culture method with the MEA technology. We have developed a full 3D neural tissue culture process and a data acquisition system composed of high-end electronics and novel MEA biochips based on porous, flexible, thin-film membranes integrating recording electrodes, named as “Strip-MEA,” to allow the maintenance of an ALI around the 3D neural tissues. The main motivation of the porous MEA biochips development was the possibility to monitor and to study the electrical activity of 3D neural tissues under different recording configurations, (i) the Strip-MEA can be placed below a tissue, (ii) or by taking advantage of the ALI, be directly placed on top of the tissue, or finally, (iii) it can be embedded into a larger neural tissue generated by the fusion of two (or more) tissues placed on both sides of the Strip-MEA allowing the recording from its inner part. This paper presents the recording and analyses of spontaneous activity from the three positioning configurations of the Strip-MEAs. Obtained results are discussed with the perspective of developing *in vitro* models of brain diseases and/or impairment of neural network functioning.

## 1 Introduction

Three dimensional (3D) neural tissues (i.e., brain slices, organotypic cultures, neurospheres, and brain organoids) offer a battery of powerful technologies to study the complex neuronal activity that emerges from neural networks capable of reproducing some features of the developing human brain (Sundstrom et al., 2005, 2012; Frega et al., 2014; Benito-Kwiecinski and Lancaster, 2020; Chiaradia and Lancaster, 2020). Micro-electrode array (MEA) technology has been used since many years as a non-invasive method to monitor the electrophysiological activity of dissociated neuronal cultures and acute tissues *in vitro* over time (Stett et al., 2003; Steidl et al., 2006; Forro et al., 2021). Therefore, this technology is particularly appropriate to study neural networks since single action potentials can be detected across multiple electrodes on the array and deliver a precise mapping of functioning and communication throughout a population of neurons. However, electrophysiological assessment of 3D neural systems has been technically challenging. In the last decades, technological efforts have been made to develop MEA arrangements able to map the electrophysiological activity from 3D networks in 2D and 3D space (Heuschkel et al., 2002; Frega et al., 2014; Bastiaens et al., 2018; Kireev et al., 2019; Soscia et al., 2020; Passaro and Stice, 2021; Shin et al., 2021; Huang et al., 2022; McDonald et al., 2023; Yang et al., 2024). An important constraint on the culture of engineered 3D neural tissues is the long time necessary to obtain sufficient network maturation within MEA biochips to be able to record neuronal activity (typically several weeks to months of culture) making the overall experimental process long lasting and material intensive before recording electrophysiological activities. One culturing approach and experimental set-up for such 3D tissues overcoming this constraint is the Air-Liquid Interface (ALI) technique (Stoppini et al., 1991), where the tissues are not completely immersed into nutritive medium, but remain at an ALI to provide sufficient oxygenation for long-term tissue survival, with the tissues being fed from the bottom through a porous membrane (Preynat-Seauve et al., 2009; Giandomenico et al., 2019). Under these conditions, tissues can then, after maturation is reached, be placed onto an ALI adapted MEA biochip (Thiebaud et al., 1997; Vanvliet et al., 2007) for experimental recordings/measurements (Tieng et al., 2014; Cosset et al., 2019).

In this work, we introduce a novel MEA biochip specifically designed for the functional monitoring of neuronal networks at the air-liquid interface. The key features of the presented “Strip-MEA” are (1) their very low thickness leading to flexible properties, (2) it is made of a porous polymer membrane-based substrate/interface allowing liquid and nutrient uptake via capillary forces and diffusion, (3) furthermore, the Strip-MEAs can be easily placed either at the bottom, on top, or embedded in between two 3D neural tissues. These Strip-MEAs are integrated within a small-volume MEA biochip perfusion system allowing a good long-term survival of the neural tissues at the ALI. We will present the full system and experimental results showing that the ALI technique is very well suited to study engineered neural 3D tissues for short-term as well as for long-term experiments.

## 2 Materials and methods

### 2.1 MEA-based data acquisition system

#### 2.1.1 MEA biochip design and manufacturing

**Figure 1:**
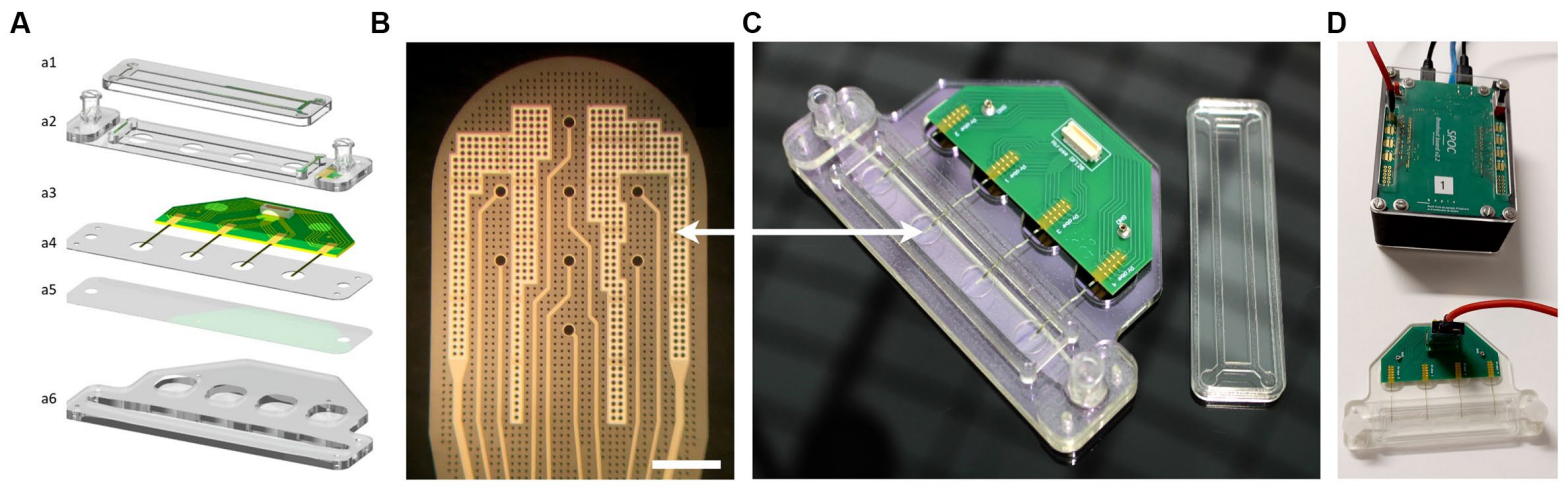
4-Strip MEA biochip and SPOC data acquisition platform. **(A)** Exploded schematic view of a 4-Strip MEA biochip; **(a1)** Lid to maintain sterility, **(a2)** Upper part with Luer Lock connector, **(a3)** PCB with Omnetics connectors, and 4 independent porous Strip-MEA probes, **(a4)** PMMA holder, **(a5)** Porous PET membrane allowing medium and gas exchange and an air-liquid interface culture, **(a6)** Bottom part of the fluidics corresponding to 2 mL of culture medium. **(B)** Enlarged view of one Strip-MEA working area. It is composed of 8 recording electrodes coated with a platinum black layer allowing low-impedance electrode characteristics. Electrodes size is a diameter of 30 μm, electrodes are located on a 200 μm grid. The light grey parts correspond to two large reference electrodes. Scale bar = 200 μm. **(C)** Image of the resulting 4-Strip MEA biochip showing the Luer Lock connectors for the inlet and outlet of the perfusion chamber underneath the working area. **(D)** Image showing the 4-Strip MEA biochip connected by a 32-channel cable to the data acquisition and pre-data analysis hardware SPOC platform (Wertenbroek et al., 2021).

The MEA biochip named “4-Strip MEA biochip” developed in this work is designed in a way that one can record simultaneously from one to four independent neural tissues in parallel where each tissue is placed on a Strip-MEA probe integrating eight recording electrodes. The designed customized 32-channel 4-Strip MEA biochip (see Figures 1A,C) is composed of a printed circuit board substrate for connection to external electronics of the data acquisition system via a board-to-board connector, a fluidic channel covered with a porous polyethylene terephthalate (PET) membrane (1 μm pore size) where the inlet and outlet enable gentle and efficient media perfusion of the 3D neural tissue from the bottom, and four Strip-MEA thin-film polyimide (PI) membranes integrating each eight platinum black recording electrodes that are placed on top of the PET membrane. Since the Strip-MEA is porous, the perforated design of the Strip-MEA allows for continuous exchange of media through the tissue sample and enables conformal contact with the neural tissues without inducing any damage.

The Strip-MEA membranes were microfabricated on 4-inch silicon wafers with a sacrificial titanium-tungsten alloy/aluminum release layer (TiW/Al, 100 nm/400 nm). A PI layer (PI2611, HD Microsystems) was spin-coated at 3,350 rpm for 40 s and then cured by a soft bake (3 min at 70°C and 3 min at 110°C) followed by a hard bake (1 h at 200°C followed by 1 h at 300°C with nitrogen from 150°C) in order to obtain a 4 μm thick PI layer. A titanium/platinum/titanium (Ti/PT/Ti, 50 nm/150 nm/50 nm) layer was then deposited by sputtering (Pfeiffer SPIDER 600 sputter cluster system). A standard positive photolithography step followed by an ion beam etching step (Veeco Nexus IBE350) allowed to shape the electrodes and their connection wires. A second 4 μm thick PI layer was then coated and hard-baked, generating the top part of the Strip-MEA membrane. A 250 nm layer of SiO_2_ was then deposited on top (Pfeiffer SPIDER 600 sputter cluster system). A standard positive photolithography step followed by dry etching (SPTS APS Dielectric Etcher) of SiO_2_ and PI with, respectively, chlorine and oxygen chemistries allowed the exposure of metal pads and electrodes as well as to shape the PI membrane and etch through holes generating porosity. Finally, a 1% HF wet chemical etching step allowed the removal of the top titanium layer of the electrodes. The Strip-MEA membranes were released by aluminum anodic dissolution (voltage of 700 mV) in saturated saline solution for 4 h. The resulting Strip-MEA membranes are composed of a flexible 8 μm thick, porous PI membrane including eight recording sites each. Electrodes have a diameter of 30 μm and are located on a 200 μm grid. Holes of ø7.5 μm on a 20 μm grid etched through the membrane generate a 10% area membrane porosity at its workspace (Figure 1B). A short video showing the flexibility of the Strip-MEA is provided in the Supplementary Data S7.

The obtained Strip-MEA were then connected to a printed circuit board using 2 component silver-epoxy glue (H20E, EPO-TEK) and sealed with epoxy (302-3 M, EPO-TEK). Platinum electrodes were sequentially coated with a layer of platinum black (300–500 nm thickness) using a platinum solution made of: 2 g H_2_PtCl_6_ xH_2_O, 16 mg C_4_H_6_O_4_Pb · 3 H_2_O, (Sigma) and 58 g H_2_O. An alternative signal at 300 Hz and 800 mV amplitude was applied via a customized home-made impedance measurement and multiplexer platform (HES-SO Coat&Check project) until the electrode impedance reached a magnitude below 8 kΩ and a phase of approximately −45°. About 10–15 s coating process are required on each electrode to achieve sufficient plating corresponding to the desired black platinum coating (see Figure 1B), resulting in a final electrode impedance below 100 kΩ measured at 1 kHz, 100 mV in 0.9% NaCl saline solution, which finally corresponds to a typical peak-to-peak noise level of approximately ±10–15 μV.

To finalize the 4-Strip MEA biochip fabrication process, the fluidic channel and connections were made out of PMMA plastic parts, a PET membrane was laser cut, and all parts were assembled using 3 M double side tape (467MP adhesive, 3 M). The final complete 4-Strip MEA biochip is shown in Figure 1C.

#### 2.1.2 Data acquisition system and data analysis

**Figure 2:**
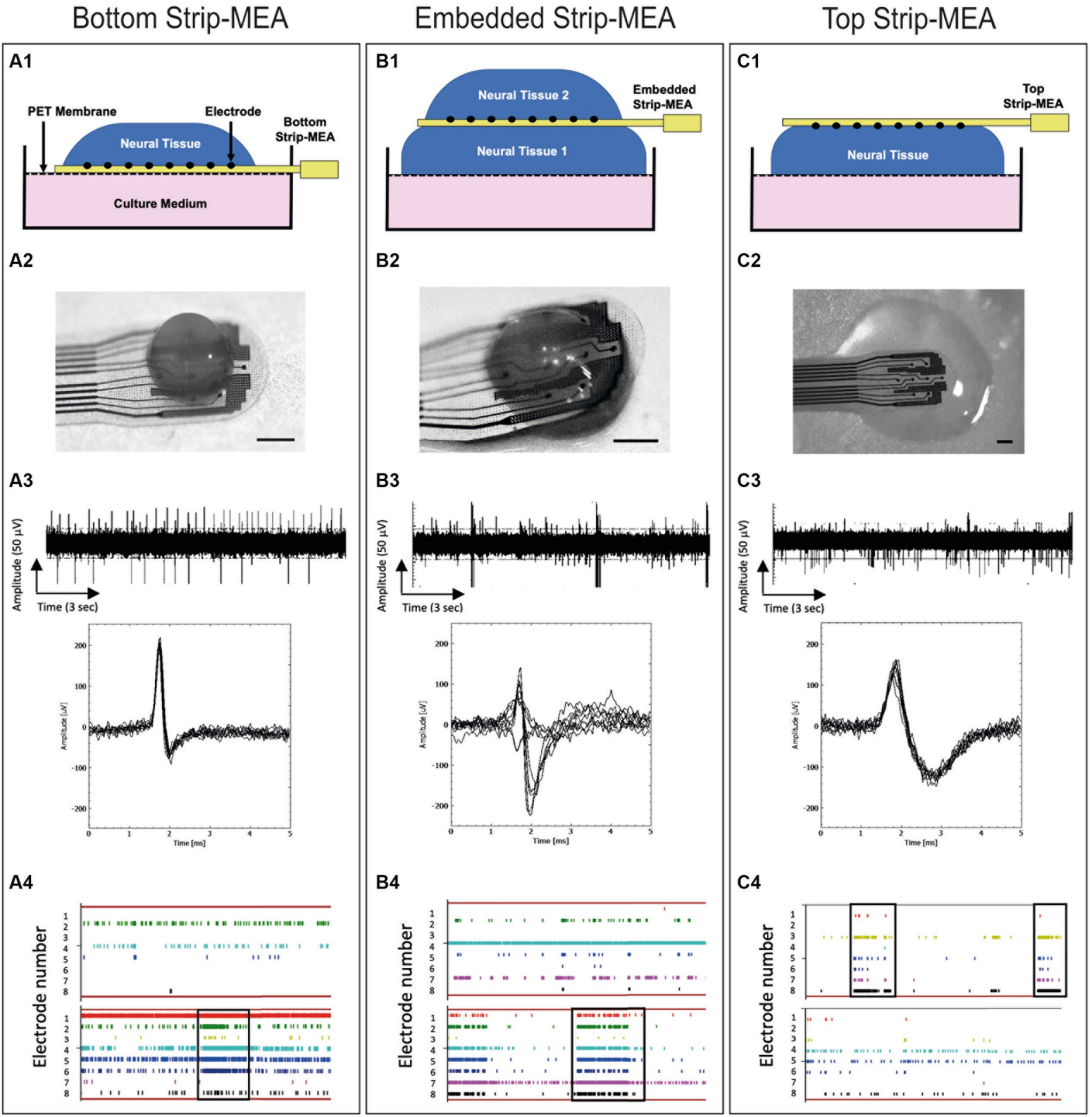
Schematic views and pictures of the different recording configurations of the Strip-MEA: **(A1)** Bottom Strip-MEA configuration: the Strip-MEA is located underneath the 3D neural tissue. **(B1)** Embedded Strip-MEA configuration: Two 3D neural tissues are placed on both sides of the Strip-MEA. **(C1)** Top Strip-MEA configuration: The Strip-MEA is located on top of the 3D neural tissue. **(A2–C2)** Pictures of 3D neural tissues and Strip-MEA in the different configurations showed in the schematic views. Scale bar = 250 μm. **(A3–C3)** Examples of raw data recorded from 17 to 18 months old 3D neural tissues during 15 s at day 3 with the MEA platform representative of spontaneous activities in the different Strip-MEA configurations with overlay spike plots (below). These raw data demonstrate the detection of action potential spikes and bursts. The amplitudes of the detected spikes are comprised between 50 and 100 μV. **(A4–C4)** Raster plots showing neural activities over time recorded from 8 electrodes at day 1 (upper) and day 15 (lower). Each bar of color represents a spike. Each line represents an electrode. The overall represented time course corresponds to 1 min. The black boxes indicate examples of synchronizations of the spike activity in one tissue.

Electrophysiological signals were acquired using the data acquisition system called “Spike-On-Chip” (SPOC) that was presented in Wertenbroek et al. (2021) (see Figure 1D). Briefly, the hardware allowing the detection of single unit action potentials and the cut-out of the activity related time-window data is time-multiplexed among 32 channels. Each channel is sampled at a frequency of 30 kHz. The amplifier bandpass filter was set from 0.1 Hz to 5.0 kHz (1st order high-pass, 3rd order Butterworth low-pass). The acquisition hardware allows the real-time detection of spikes by applying a simple threshold-crossing criterion. Voltage variations larger than six times the standard deviation are considered as spikes. Each time that the threshold is reached a 5 ms window is recorded (starting 1 ms before the event) (see Figures 2A3–C3). The signal-to-noise ratio (SNR) is calculated between two-time intervals which delimit where the waveforms vary in a non-negligible way. In general, the “low value for the standard deviation” corresponds to 0.5 mV while the “high value for the standard deviation” corresponds to 1.5 mV.

To study the functionality of the 3D neural tissues, the following parameters are analyzed off-line: Number of active electrodes, number of spikes detected by each electrode, burst detection (burst frequency: network activity), network burst detection, signal/noise, spike frequency rate (in Hz), burst duration, mean frequency of spike in bursts, amplitude of spikes from the same electrode over time. It was considered that a burst is a series of three consecutive spikes firing no more than 50 ms apart from each other. Alignment of spikes or bursts on all electrodes can be observed on raster-plots corresponding to the synchronization of neuronal activity from the 3D neural tissue (Figures 2A4–C4). Features were calculated per channel for each device.

### 2.2 3D neural tissue generation

Neural stem cells derived from induced pluripotent stem cells (NSChIPSC) (#A3890101 Thermo Fisher Scientific) were seeded with a density of 25,000 cells/cm^2^ in six-well plates and processed for the generation of 3D neural tissue according to the protocol previously described in Govindan et al. (2021). Briefly, the generation of 3D neural tissues, was obtained by detaching the cells at approximately 80% confluence with pre-warmed StemPro™ Accutase™ (A1110501, Thermo Fisher Scientific) for 1–2 min. The single cell suspension was centrifuged for 3 min at 320 g, suspended in proliferation medium and cells were counted. 500,000 cells in 3 mL proliferating medium were added into a non-treated six-well plate. The plate was left under orbital agitation (80 rpm) for 4 days in a cell culture incubator at 37°C (100% humidity, 5% CO_2_). 24 h later, the free-floating 3D neural tissues were formed by aggregation. Four days after seeding, the 3D neural tissues size was checked and switched to a differentiation medium composed of NeuralQ™ Basal Medium (GSM9420, GlobalStem), GS21T Supplement (GSM3100, GlobalStem), and 1% Glutamax™ supplement (35,050,061, Thermo Fisher Scientific). Cultures were maintained in orbital agitation (80 rpm) for 6 weeks. A breathable plate sealer was added on top of the 6-well plate reducing medium evaporation. The medium was changed once a week. Experiments can be performed with 3D neural tissues that have a minimal age of 8 weeks, as they show sufficient maturation to present electrical activity that can be recorded with the data acquisition system. However, for all the presented experiments, 17–18 months old 3D neural tissues were used. During the tissue maturation process, 3D neural tissues exhibit a steady increase in firing rate, burst frequency, synchronicity and population spiking as it was shown in previous work (Trujillo et al., 2019).

### 2.3 Description of air-liquid interface principle

ALI cultures were originally developed for organotypic cultures of brain slices, and it has been shown to retain many essential organizational features of the host tissue (Stoppini et al., 1991; Sundstrom et al., 2005). An important feature of the ALI culture is the improved exchange between air and tissue, allowing the development of a relatively thick 3D culture without hypoxic cell death. In fact, by addition of the culture medium only underneath a porous membrane, the neural tissues lying on top of this membrane are covered by capillarity by a very thin film of medium, allowing important air diffusion within the entire thickness of the tissues.

In the presented experiments, mature (17–18 months old) 3D neural tissues have been transferred onto ø6 mm patches of pre-cut circular hydrophilic membrane supported by a six well insert (PICMORG50, Merck-Millipore) at air/liquid interface. 3D neural tissues can be left in these conditions for several days up to several months if needed. The use of membrane pre-cut patches facilitates the tissue manipulation for further imaging or electrophysiology experiments (Preynat-Seauve et al., 2009; Ferlauto et al., 2018). It results that the previously described 3D neural tissues culturing technique allows the generation of many mature 3D neural tissues ready to be transferred onto MEA biochips for electrophysiological experiments. This approach represents an interesting advantage since it is not necessary to culture the tissues directly on the MEA biochips for a long period of time prior to recordings. Electrical activity can therefore be recorded shortly after placing the tissue in contact with the Strip-MEAs (Figure 2).

#### 2.3.1 Neural tissue positioning and recording protocol

Mature neural tissues were positioned onto ø6 mm pre-cut patches of membrane and were then transferred with forceps under microscope either onto or below a Strip-MEA or on both sides of the Strip-MEA (see Figures 2A1–C1).

Spontaneous activities were recorded for 30 min after tissue deposition (2 h and 6 h after deposition) and subsequent days up to 3 weeks. For the different Strip-MEA configurations, experiments were performed using three 4-Strip MEA biochips having each four independent Strip-MEAs covered with tissues.

#### 2.3.2 MEA data analysis: spike sorting

**Figure 3:**
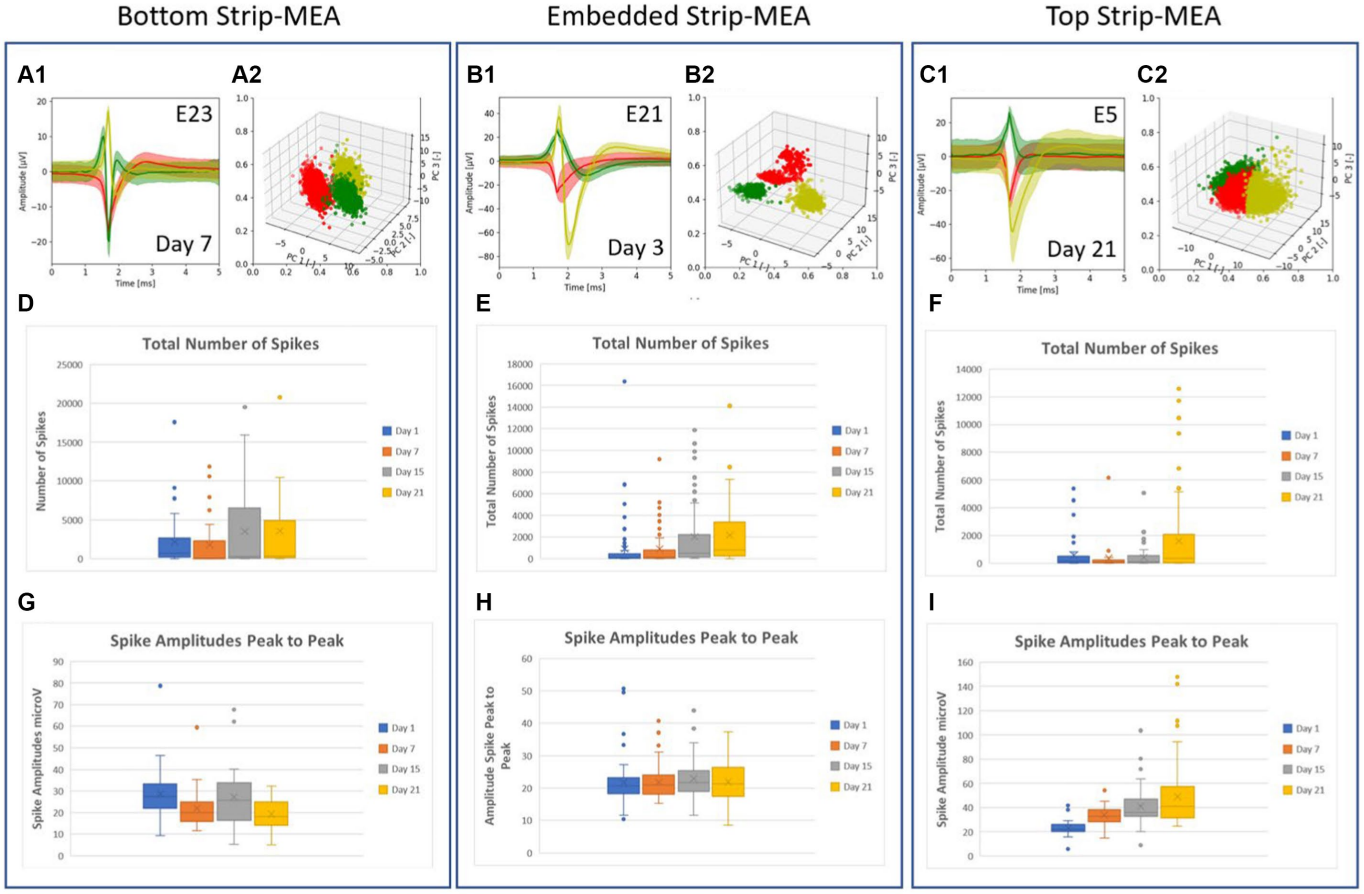
**(A–C)** Example of typical traces of the different waveforms recorded from one electrode after PCA spike sorting analysis and the related graphs of point clouds where each point represents a spike and each color represent a cluster. Each cluster or unit represents the activity of a single neuron in the 3D neural tissue. Most of the time, 1, 2 or 3 spikes coming from different neurons can be recorded at an electrode. **(D–F)** Boxplots showing the total number of spikes recorded at days 1, 7, 15, and 21. **(G–I)** Boxplots showing amplitudes of spikes peak-to-peak at days 1, 7, 15 and 21. All values used in the boxplots correspond to number of spikes and amplitudes of spikes, counted, and measured per individual electrodes during a time frame of 30 min, respectively.

Detected spikes were processed by using principal component analysis (PCA), which separates the different waveforms found in the data. We are using the sklearn.decomposition for the PCA data analysis with the following parameters: the starting point is set at 1.4 ms and the end is set at 2.4 ms. It is creating a range between the start and the end for the PCA analysis of waveforms/spikes. Those parameters were chosen because in this time range, the waveforms vary significantly leading to a better cluster separation in the PCA space. The sorted spikes are then grouped into clusters based on the similarity of their shapes (see Figures 3A1–C1). A point of cloud graph is also generated where each point represents a spike and each color a cluster (see Figures 3A2–C2). This spike sorting analysis determines if signals recorded from one electrode are coming from a single or a group of neurons.

### 2.4 Imaging

#### 2.4.1 Sample preparation

The imaging experiments were performed using 3D neural tissues in contact with Strip-MEAs during 19 days in culture. All samples were then transferred into phosphate buffer (0.1 M, pH 7.4) containing 4% formaldehyde and 2.5% glutaraldehyde. Fixation is done at room temperature for 2 h after which the samples are stored in fresh fixative overnight at 4°C.

After fixation, the samples are sequentially post stained in 4% osmium aqueous solution for 45 min in the dark and in 2% uranyl acetate aqueous solution for 1 h. Then, samples are dehydrated in a series of ethanol concentrations, ranging from 10 to 100% then infiltrated with EPON resin (Electron Microscopy Sciences, USA) before polymerization at 60°C for 24 h. Thin sections of 90 nm were cut and mounted onto a Formvar film-coated, carbon-stabilized 200 mesh copper grid (Electron Microscopy Sciences, USA). The sections are post stained with 2% uranyl acetate aqueous solution for 15 min then lead citrate for 3 min.

For scanning electron microscopy (SEM) preparation, samples were fixed and dehydrated as described previously. After the final step in ethanol 100%, samples were critically dried with an EM CPD300 (Leica Microsystem, Germany).

For SEM imaging, resin block faces and/or the samples are coated with a 7.5 nm layer of a mix of gold and platinum, obtained using a gold (40%) platinum (60%) target, and mounted on an aluminum pin (Electron Microscopy Sciences, USA) stabilized with carbon tape and conductive silver glue.

#### 2.4.2 TEM imaging

For TEM imaging, thin sections were imaged with a Tecnai-12 transmission electron microscope (Thermo Fisher Scientific, USA) operating at 120 kV with a bottom mount F416 camera (4Kx4K) (TVIPS, Germany) using EM-Menu software (TVIPS, Germany).

#### 2.4.3 SEM imaging

For SEM imaging, resin blocks were imaged with a Schottky Field Emission scanning electron microscope SU5000 (Hitachi, Japan) operating with the EM Wizard interface (Hitachi, Japan).

## 3 Results

### 3.1 Human iPSCs-derived neurons aggregate into 3D neural tissues

**Figure 4:**
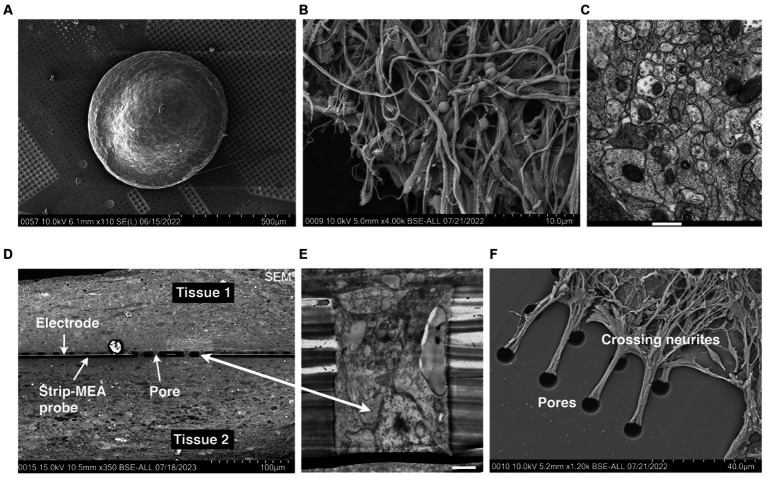
**(A)** Scanning electron microscopy (SEM) microphotography showing a 3D neural tissue laying down on top of a porous Strip-MEA. **(B)** SEM microphotography showing a 3D neural tissue at higher magnification. **(C)** Transmission electron microscopy (TEM) of a transversal section of a 3D neural tissue showing a compact parenchyma. Scale bar = 500 nm. **(D–F)** Histological visualization of the integration/fusion of a Strip-MEA probe when placed in-between two 3D neural tissues. **(D)** SEM transversal section of the embedded Strip-MEA probe. **(E)** TEM picture of a Strip-MEA probe pore filled with neural tissue. Scale bar = 2 μm. **(F)** SEM picture of neurites under mechanical tension crossing probe pores from a tissue embedded Strip-MEA after small tissue displacement.

We monitored the formation of 3D neural tissues over time in culture wells in an orbital shaker at 80 rpm and culture for 7 days at 37°C and 5% CO_2_. We observed that hiPSCs-derived neuroprogenitors started aggregating after 24 h and an increase in size is visible after 7 days due to proliferation. The aggregates showed a spherical shape with a mean diameter between 500 and 700 μm after 6 months and remain this size after 1 to 2 years in orbital cultures. In previous studies, we have shown that these 3D neural tissues are mature because the tissues express protein and gene differentiation markers as presented in Figure 4 of Govindan et al. (2021). 3D neural tissues were then transferred onto pre-cut patches of membranes and placed in cell culture inserts to facilitate their manipulation for further experiments. The spheroid shape of the tissues in rotation is changed when seeded on a planar surface. As shown in Figure 4, the diameter of the 3D neural tissue on porous MEA is within the range of 500–700 μm and its thickness is within a range of 150–250 μm.

### 3.2 4-Strip MEA biochip and electrophysiological platform

We have designed and manufactured a 4-Strip MEA biochip which allows the maintenance of 3D neural tissues at the ALI for electrophysiological recordings. The biochip can be placed within the incubator and connected to the SPOC data acquisition system by a cable (see Figure 1D). The flexible ultrathin Strip-MEA can be positioned in three configurations: (1) Bottom Strip-MEA: where the Strip-MEA is located underneath the 3D neural tissue (Figure 2A1), (2) Embedded Strip-MEA: where two 3D neural tissues are placed on both sides of the Strip-MEA (Figure 2B1), and (3) Top Strip-MEA: where the Strip-MEA is located at top of the 3D neural tissue (see Figure 2C1).

### 3.3 Characterization strip-MEA electrophysiology

We have investigated the electrophysiological activities of the 3D neural tissues when the Strip-MEAs were positioned at different 3D neural tissue locations. In the three configurations we could record spontaneous activities already 2 h after the placing of the neural tissues in contact with the Strip-MEA. We followed-up these activities during a period of 3 weeks. Examples of representative raw data of active neurons are shown in Figures 2A3–C3 at 3 days after the beginning of the experiment. Inserts show the superposed spikes detected from one electrode in each configuration. Example of raster plots of timestamps show the spike activity during recording sessions after 1 and 15 days in contact with the Strip-MEAs (see Figures 2A4–C4). It can be noticed that burst activities as well as network synchronization in the three configurations are present. In most of the experiments, we found that the number of active electrodes and spike firing tend to increase over time as shown in raster plots (see Supplementary Data S1).

An example of neuronal activity summary at day 21 from 8 electrodes from one embedded Strip-MEA is shown in Table 1. Main neural activities were analyzed for spiking, bursting and a unit analysis using PCA spike sorting was also performed. In this example, we could detect spikes from the 8 electrodes with a quite large distribution. On the other hand, burst activities could not be observed in two electrodes (E19, E21). Following the data presented in Table 1, spike sorting revealed that most of the electrodes were recording 2 different shapes of spikes (named U1 and U2 for Unit 1 and Unit 2), suggesting that the electrodes were recording activity from 2 different neurons, and even from up to 3 neurons on the electrode E24 (U1, U2 and U3).

**Table 1:** Example of data analysis: spike and burst activities from 8 electrodes from a single embedded Strip-MEA out of 4 from one 4-Strip MEA biochip.

		**Spike analysis**				**Burst analysis**					**Spike sorting**			
**Strip ID**	**Electrode ID**	**Mean frequency [Hz]**	**SNR mean [amp PTP/STD noise]**	**Mean amplitude Peak to Peak [μV]**	**Total number spikes**	**Number of burst**	**Frequency of burst [min** ^**−1**^ **]**	**Burst duration mean [s]**	**Mean number of spikes in burst**	**Mean spike frequency in burst [Hz]**	**Number of units sorted per electrode**	**Total number of spike**	**Mean frequency [Hz]**	**Pattern**
Strip-MEA N° 2	E17	4.1	9.9	81.6	7,356	69	2.30	0.1	7.4	68.3	U1	2,770	1.54	Bursting
											U2	4,585	2.55	Bursting
	E18	10.6	8.9	65.9	19,006	625	20.83	0.1	9.1	78.7	U1	14,129	7.85	Bursting
											U2	4,876	2.71	Bursting
	E19	0.1	7.1	78.3	181	0	0.00	0.0	0.0	0.0	U1	140	0.08	Bursting
											U2	40	0.02	Slow
	E20	8.8	10.3	77.8	15,848	185	6.17	0.1	7.7	68.9	U1	8,520	4.73	Irregular
											U2	7,327	4.07	Bursting
	E21	1.9	8.7	62.7	3,441	0	0.00	0.0	0.0	0.0	U1	2,615	1.45	Irregular
											U2	825	0.46	Bursting
	E22	1.3	9.1	84.7	2,372	13	0.43	0.1	6.8	67.7	U1	2,167	1.2	Bursting
											U2	204	0.11	Bursting
	E23	5.5	8.3	59.2	9,917	101	3.37	0.1	7.6	69.7	U1	6,161	3.42	Bursting
											U2	3,755	2.09	Bursting
	E24	9.3	10.4	72.9	16,736	363	12.10	0.1	7.9	70.2	U1	4,393	2.44	Irregular
											U2	5,684	3.16	Bursting
											U3	6,657	3.7	Bursting

Spike activity parameters presented are: mean frequency; mean signal to noise ratio (SNR mean: spike peak-to-peak amplitudes are considered, divided by standard deviation of the noise); mean amplitude of the spikes; total number of spikes. Burst activity parameters are: number of bursts; frequency of burst per minute; burst duration in seconds; mean number of spikes in bursts; mean spike frequency in burst in Hz. Spike sorting analysis was performed to get unit analysis (different spikes detected on the same electrode), total number of spikes, mean frequency of spikes in Hz and the pattern of activity (slow, bursting, irregular).

A spike sorting analysis of the recorded data from one 4-Strip MEA biochip in the embedded Strip-MEA configuration was also performed (corresponding to four independent 3D neural tissues). Figures 3A1–C1 show examples of traces of the different waveforms after spike sorting analysis from each raw data electrode represented in Figures 2A3–C3. The graphs of point clouds (Figures 3A2–C2) represent each spike by a point and each color represents a cluster. We could observe that some electrodes could record up to three different waveforms (Figure 3C1).

The quantitative analysis from three biochips where the four Strip-MEAs are located either at the bottom, embedded or on top of neural tissues are shown in Figure 3 (data from all experiments carried out in this work are provided in the Supplementary Data S2). The total number of spikes recorded at days 1, 7, 15, and 21 in each recording configuration are represented by boxplots (see Figures 3D–F) and boxplots in Figures 3G–I, show peak-to-peak amplitudes of spikes at days 1, 7, 15, and 21. The burst activities in the same batch were also analyzed at days 1, 7, 15, and 21 (see Supplementary Data S3).

The analyses from this series of *N* = 3 experiments (corresponding to 12 independent 3D neural tissues) for each of the three configurations show that we could monitor neuronal activities from the 3D neural tissues, but we could not detect a clear difference depending on the position of the Strip-MEA. The monitoring of 3D neural tissues from embedded Strip-MEA for a period of weeks shows that the shape of the single-unit spike traces remains similar over time since we could follow-up spike units showing the same shapes after PCA analysis during recording sessions at day 7, 15, and 21 (see Supplementary Data S4). These results are encouraging since we were wondering if the presence of the Strip-MEA might impair the survival of the surrounding neural tissue close to the Strip-MEA. The fact that the Strip-MEA probe presents a 10% membrane area porosity allows a good anchorage of the tissues through the porosity (see Figure 4F), which is certainly an important factor for the mechanical stability of the tissue on the Strip-MEA. Moreover, the porosity also provides space for the neurites to cross the Strip-MEA membrane, which allows the fusion and communication between two neural tissues located on both sides of the Strip-MEA as it can be observed at scanning electron microscopy (see Figures 4D,E).

### 3.4 Histology of a strip-MEA embedded within two 3D neural tissues

In a first series of experiments, we looked at SEM level 3D neural tissues laying down on top of a porous Strip-MEA (see Figure 4A). At higher magnification compact neurites can be observed either at SEM (Figure 4B) or TEM (Figure 4C) levels. The integration of the Strip-MEA probes, in the embedded configuration was also investigated by electron microscopy (SEM and TEM) after 19 days of culture in contact with the Strip-MEA probe. We performed a series of images to reveal the fusion of the two neural tissues around the Strip-MEA probe (see Figure 4D). At higher magnification (TEM level) we could visualize that neural tissues were filling up the pores of the Strip-MEA membrane (see Figure 4E). Finally, a SEM picture showing neurites crossing the Strip-MEA pores to connect two neural tissues is presented in Figure 4F. This study confirms the good survival and communication between the two neural tissues in the embedded the Strip-MEA configuration.

## 4 Discussion

Electrophysiology is in fact one of the most sensitive and neuronal specific endpoints to assess functional neural activity. It provides high-information content of the neuronal tissue’s functional behavior. In the early 80’s, advancements in microfabricated technologies enabled the introduction of a new generation of devices, Micro-Electrode Arrays (MEA), which allowed multi-site, long-term recordings of the electrical activity of neuronal populations as well as the stimulation from one or more electrodes of the array (Seidel et al., 2017). MEA-based neuro-electronic interfaces are now a well-accepted technique in basic and applied electrophysiology, enabling experimental investigations of collective dynamics, spatiotemporal patterns and computational properties of neuronal assemblies in manners that were inaccessible before (Frega et al., 2014; Obien et al., 2015; Forro et al., 2021; Tanwar et al., 2022). The ability to monitor the functional dynamics of the entire 3D reconstructed neural tissue is a critical bottleneck (Soscia et al., 2020; Choi et al., 2021) and inserting MEA probes into 3D neural tissue is a complex challenge, and work is ongoing to develop new generations of suitable (more flexible) probes (Kireev et al., 2019; Park et al., 2021; Sharf et al., 2022; Cai et al., 2023; Lam et al., 2023; McDonald et al., 2023; Morales Pantoja et al., 2023; Yang et al., 2024). 3D neural tissues are extremely sensitive to experimental conditions and require adequately designed MEA devices that allow the maintenance of an air-liquid interface around the 3D neural tissue during culture and recording (McDonald et al., 2023). To achieve a good survival of 3D neural tissues on solid MEAs, the main strategy is to lay down brain spheroids or organoid slices onto pre-coated substrates and to add only few drops of culture medium until the attachment of the tissues to the electrodes. In this configuration, the tissues are at air/liquid interface. In a second step, additional culture medium can be added to the MEA culture plate to completely submerge the neural tissues. The main issue with this approach might be the diffusion of oxygen and culture medium in the center of the tissue leading to necrotic cores (Fair et al., 2020; Schröter et al., 2022; Sharf et al., 2022).

In the present study, we have introduced a novel air/liquid ultrathin flexible and porous Strip-MEA to analyze 3D neural network activity. In this approach, only a thin film of culture medium covers the tissue allowing a good gas diffusion within the entire thickness of the tissue preventing the formation of a necrotic core. There is no need for any MEA surface treatment (adhesion promoting coating) to “glue” the tissue to the MEA to insure good coupling of the MEA electrodes with the 3D neural tissues. The Strip-MEA probe used in this work provides good electrical contact due to the porosity induced capillary forces present that plate and hold in place the 3D neural tissue onto the MEA surface (see Figure 4).

The developed 4-Strip MEA biochips allow recordings not only from external sides (bottom and top) but also from the inside of 3D neural tissues (embedded configuration). Since we can detect action potentials within a very short time (sometimes only several minutes) after tissue placement, we can therefore select tissues which are responding and rapidly discard non-active 3D neural tissues before starting long-term experiments. During the validation process of this new approach, we analyzed different parameters to describe the general neuronal activity, synchronicity, as well as burst structures of multiple spike trains to confirm that the functionality of neural tissues was similar to previous works using different types of MEA devices (Muzzi et al., 2023). In addition, the “embedded” configuration could allow chimeric assemblies which may be of interest, i.e., different tissues being positioned on either side. In a previous study using a similar air/liquid porous MEA, we used pharmacological approaches and were able to illustrate and validate the functionality of the SPOC data acquisition system by injecting bicuculline, a molecule known to mimic epileptic activity on neural tissues and thus increasing bursting activity (Wertenbroek et al., 2021). In order to test the suitability of the present ALI approach in neurotoxicology, we have tested the effect of 2.5 μM trimethyl-tin (TMT), a known neurotoxic molecule, during 24, 48, and 72 h of exposition to 20-month-old 3D-neural tissues. We could observe a clear decrease of spikes as well as burst activity already after 24 h (see Supplementary Data S5).

We have also performed preliminary investigations to develop a method for identifying spikes from our MEA collected data using deep learning technology. We have designed three deep learning models, based on an auto-encoder architecture, followed by several classifiers based on implemented and personal algorithms. The best solution found so far is a dense (fully connected) auto-encoder, followed by a multi-layer convolutional neural-network based classifier to extract temporal and frequency features and perform classification of the biological signal. Briefly, the spike shape data analysis follows the following procedure. The training dataset is based on a dataset containing biological spikes that have been machine-labeled. Labeling is performed if the spike exceeds 6 times the standard deviation from noise. From there, we took our reference dataset (another dataset) and labeled it using the same technique. This data set contains machine-labelled spikes and noise taken at random from the experiment. Our neural network is based on this dataset to learn how to differentiate a spike from noise. We did the same procedure on another experiment in order to test the dataset and to know as closely as possible the number of spikes and the number of detected noises in this test dataset. We submitted the dataset to the deep learning system and analyzed the results. We obtained more than 99% accuracy (see Supplementary Data S6). Future steps to improve this analysis will be the implementation of deep learning models to classify normal versus abnormal neuronal behavior (e.g., normal condition versus experimental condition or no control versus treated condition).

## 5 Conclusion and perspectives

The novel multifunctional Strip-MEA probes introduced in this work have proven to be advantageous, versatile, and simple for many electrophysiological measurements. The porous PI structure of the Strip-MEA probes allows a good integration within neuronal tissues, resulting in detection of spike activity within a few minutes. The Strip-MEA probes are biocompatible and relatively easy to fabricate. Mature neural tissues can be grown separately and placed directly in contact with the Strip-MEA probes and electrophysiological activities can be recorded within a few minutes to a few hours after tissue placement giving the opportunity to perform quick experiments to assess the functionality of 3D neural networks. The possibility to place recording electrodes inside 3D tissues without signal degradation is a key result as it may open up the possibility to work with thicker neural tissues by aggregation, and to combine different tissue types into a larger 3D brain model (co-cultures). Stacking of more 3D neural tissues layer will be the next development towards more complex 3D brain models. In the present report, experiments were carried-out during 3–4 weeks. However, results from other experiments not shown here allowed to continuously monitor electrophysiological activity of 3D neural tissues during up to 3 months using our ALI platform. This work was performed using 3D neural tissues, but preliminary experiments have been carried out using brain organoids with similar results.

In conclusion, we expect our ALI-MEA system to open-up exiting opportunities for studies of neural circuits by investigating functional neuronal networks in 3D neural models.

## Data availability statement

The raw data supporting the conclusions of this article will be made available by the authors, without undue reservation.

## Ethics statement

Ethical approval was not required for the studies on humans in accordance with the local legislation and institutional requirements because only commercially available established cell lines were used.

## Author contributions

LS: Funding acquisition, Writing – original draft, Validation, Methodology, Investigation, Formal analysis. MH: Funding acquisition, Writing – review & editing, Methodology. CL-F: Investigation, Writing – review & editing, Methodology. LG: Writing – review & editing, Methodology. AR: Supervision, Funding acquisition, Writing – review & editing, Resources, Project administration.
