# Supplementary material for: Versatile micro-electrode array to monitor human iPSC derived 3D neural tissues at air-liquid interface
Source: Front Cell Neurosci. 2024 May 9;18:1389580. doi: 10.3389/fncel.2024.1389580 (PMC11112036; doi:10.3389/fncel.2024.1389580)
Supplement: Supplementary file 2 [file Data_Sheet_1.pdf]

Versatile micro-electrode arrays to monitor human iPSC derived 3D neural tissues at air-liquid interface

Luc Stoppini, Marc O. Heuschkel, Céline Loussert-Fonta, Loris Gomez Baisac and Adrien Roux

Tissue Engineering Laboratory, HEPIA HES-SO University of Applied Sciences and Arts Western Switzerland, Geneva, Switzerland

Supplementary Material

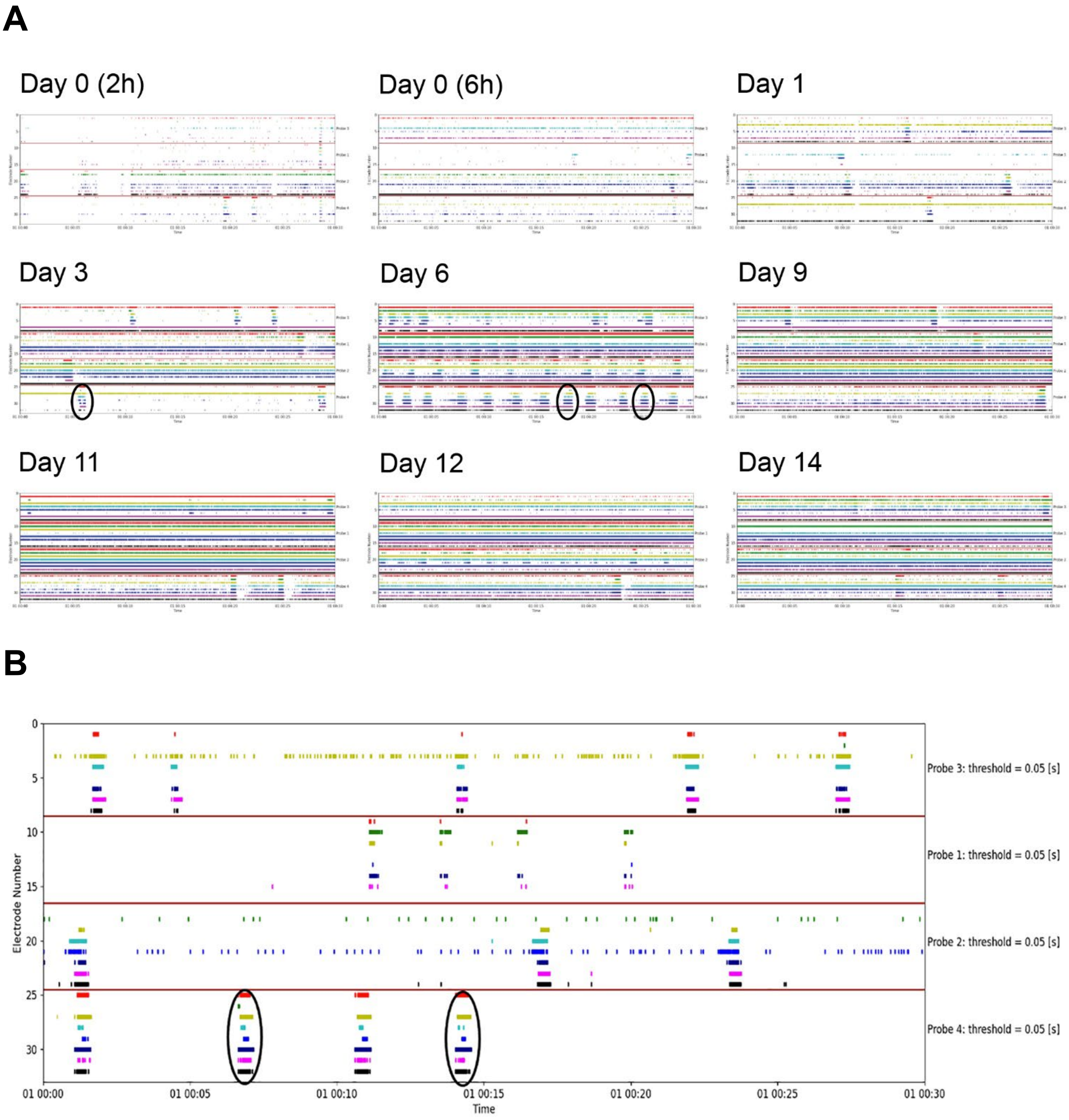

Supplementary Data S1: Time course analysis of the spontaneous activity from 17–18 months old (at start of the experiment) 3D neural tissues at embedded Strip-MEA configuration over a time course of 2 weeks. **A**) Raster plots of spike activity over time. Each colored line represents an electrode (32 electrodes at all corresponding to parallel recordings from 4 independent 3D neural tissues with 8 electrodes each). Each bar represents a spike. The black circles indicate examples of network synchronizations in one tissue. **B**) Raster plot showing only bursts at day 8.

A

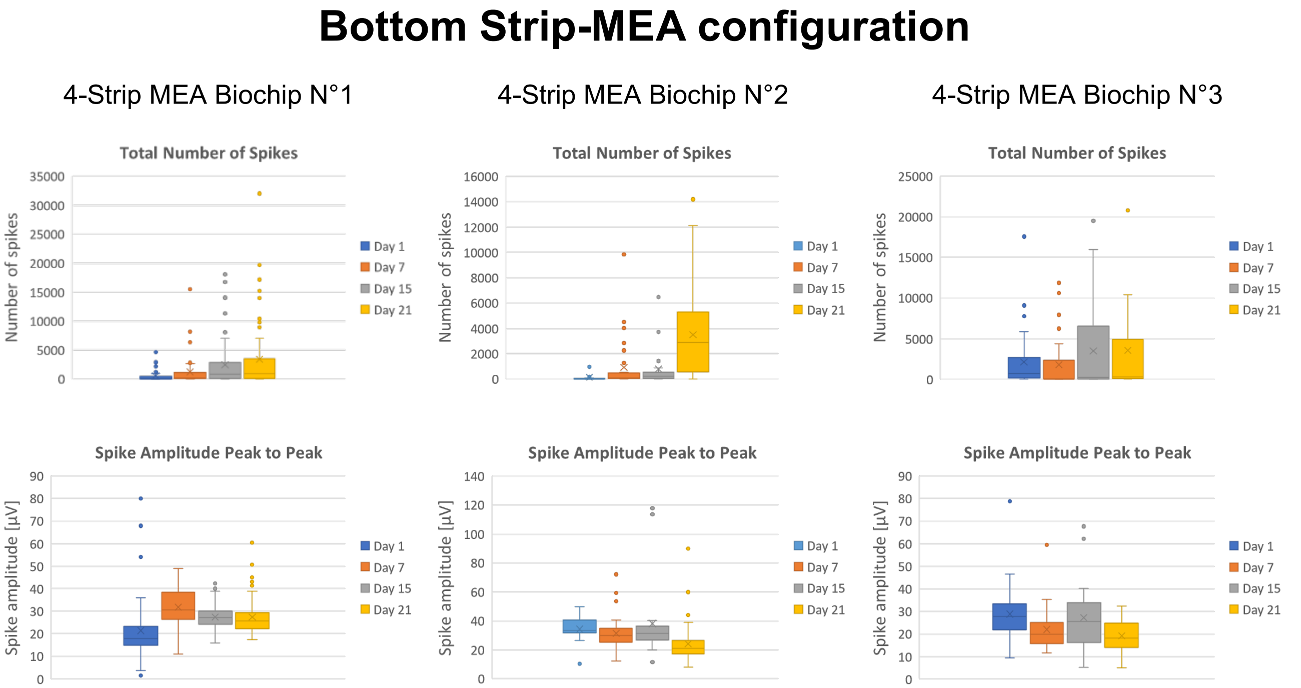
Bottom Strip-MEA configuration
4-Strip MEA Biochip N°1
4-Strip MEA Biochip N°2
4-Strip MEA Biochip N°3
Total Number of Spikes
Number of spikes
Spike Amplitude Peak to Peak
Spike amplitude [µV]
Day 1
Day 7
Day 15
Day 21

B

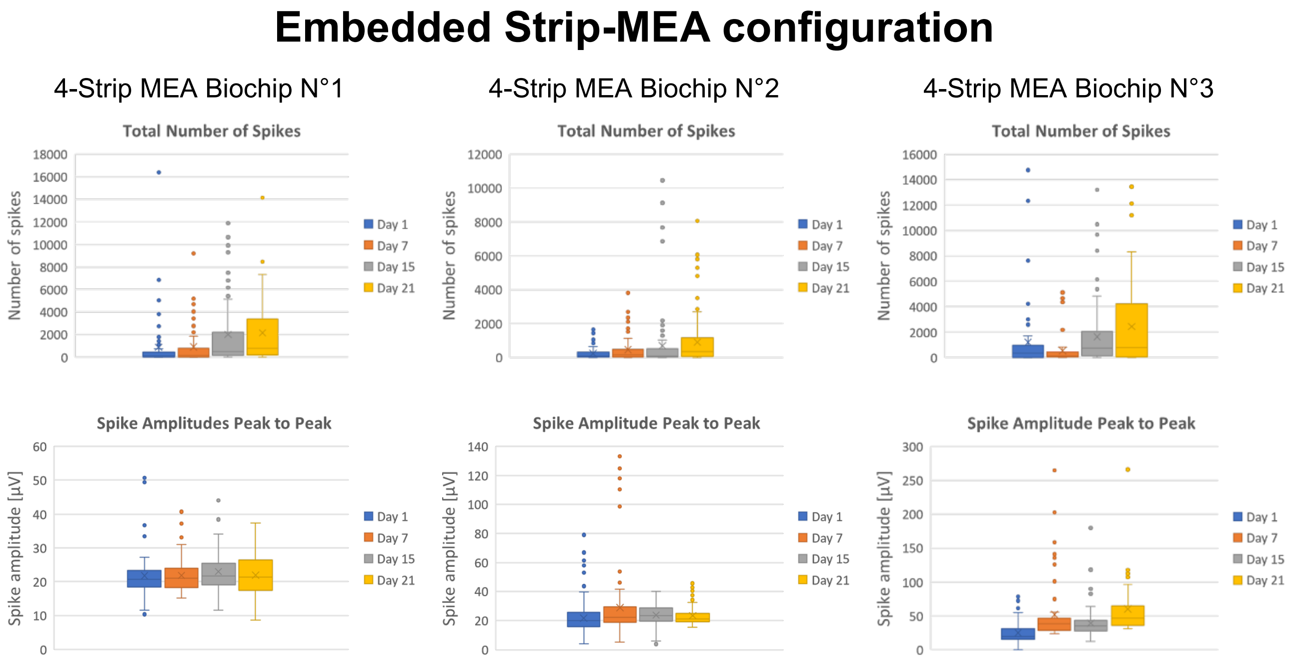
Embedded Strip-MEA configuration
4-Strip MEA Biochip N°1
4-Strip MEA Biochip N°2
4-Strip MEA Biochip N°3
Total Number of Spikes
Number of spikes
Spike Amplitudes Peak to Peak
Spike Amplitude Peak to Peak
Spike amplitude [µV]
Day 1
Day 7
Day 15
Day 21

C

Top Strip-MEA configuration

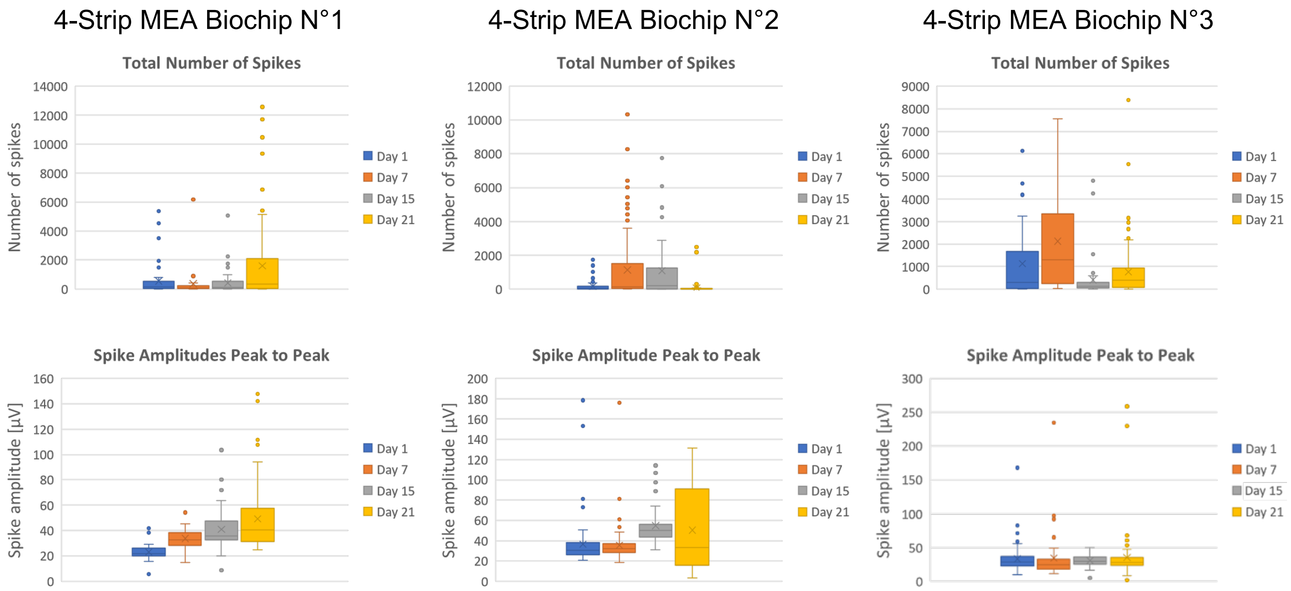

Supplementary Data S2: Experimental data from all the 4-Strip MEA biochips recordings (N=3 for each configuration) of this work using 17-18 months old (at start of the experiment) 3D neural tissues for the **A)** Bottom Strip-MEA configuration, **B)** Embedded Strip-MEA configuration, and **C)** Top Strip-MEA configuration. Each 4-Strip MEA biochip did make parallel recordings from 4 independent 3D neural tissues with 8 electrodes each. Total number of spikes and spike amplitudes from each 4-Strip MEA biochip is displayed in boxplots at days 1, 7, 15 and 21. All values used in the boxplots correspond to number of spikes and amplitudes of spikes peak-to-peak counted and measured per individual electrodes of the 4-Strip MEA biochip during a time frame of 30 minutes, respectively.

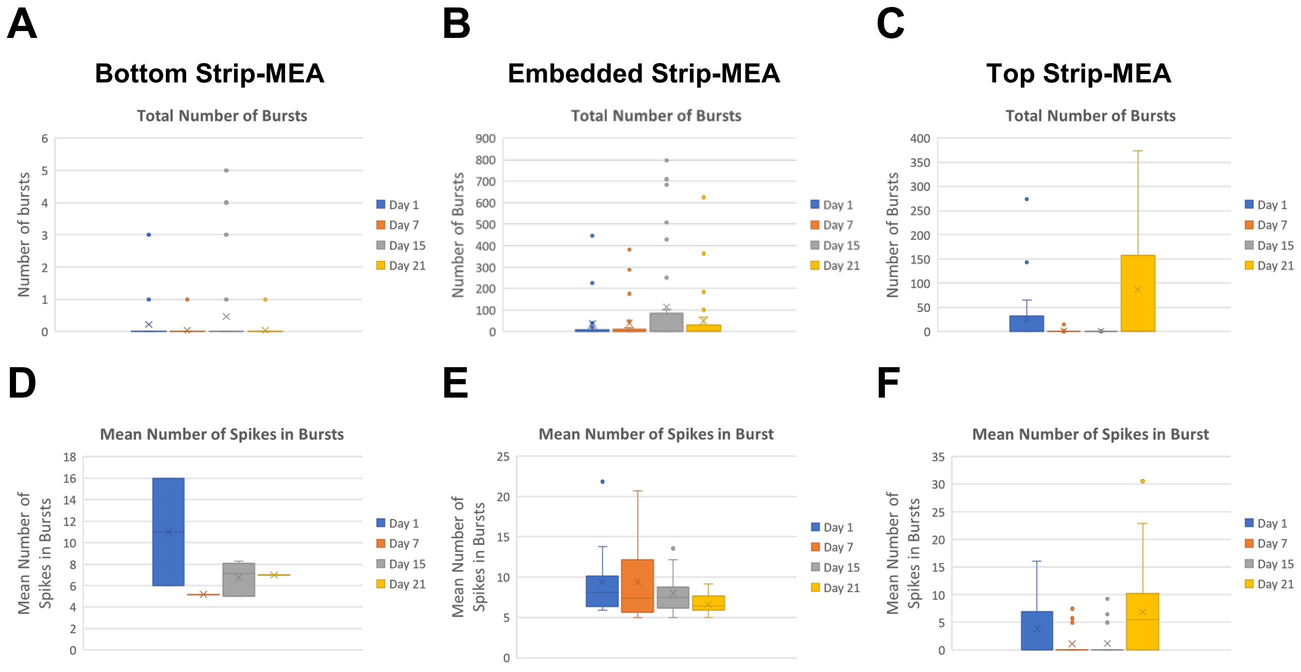

Supplement Data S3: Results of burst analysis from recordings from one 4-Strip MEA biochip at each configuration over a time course of 3 weeks. **A-C**) Boxplots showing the total number of bursts recorded from 17-18 months old (at start of the experiment) 3D neural tissues at days 1,7, 15 and 21 for the **A** Bottom Strip-MEA configuration, **B** Embedded Strip-MEA configuration, and **C** Top Strip-MEA configuration. **D-F**) Boxplots showing the mean number of spikes in related bursts at days 1, 7, 15 and 21 for the three configurations.

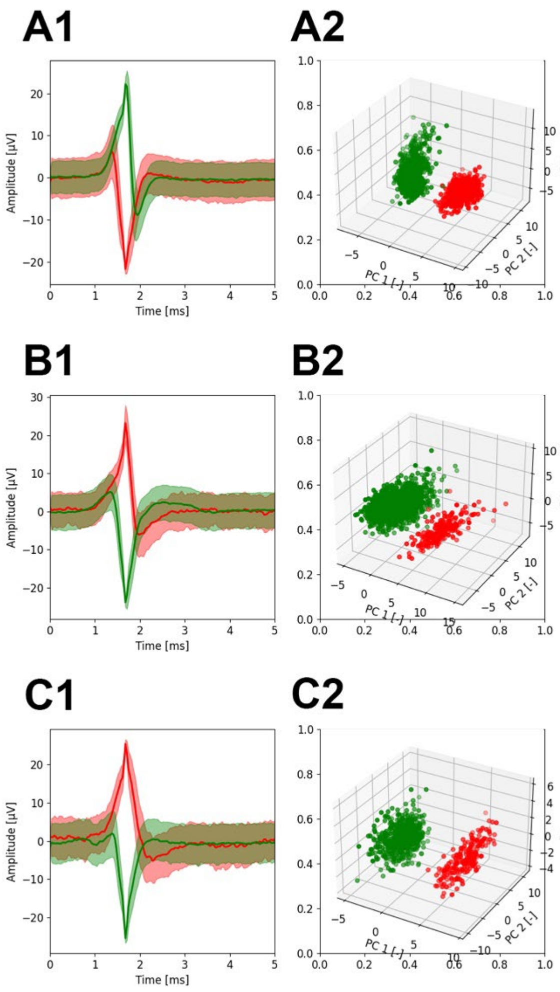

Supplementary Data S4: A-C) Traces and graph points clusters of the different waveforms after (PCA) spike sorting analysis at day 7 (**A1, A2**), day 15 (**B1, B2**) and day 21 (**C1, C2**) from the same electrode 31 from one embedded Strip-MEA.

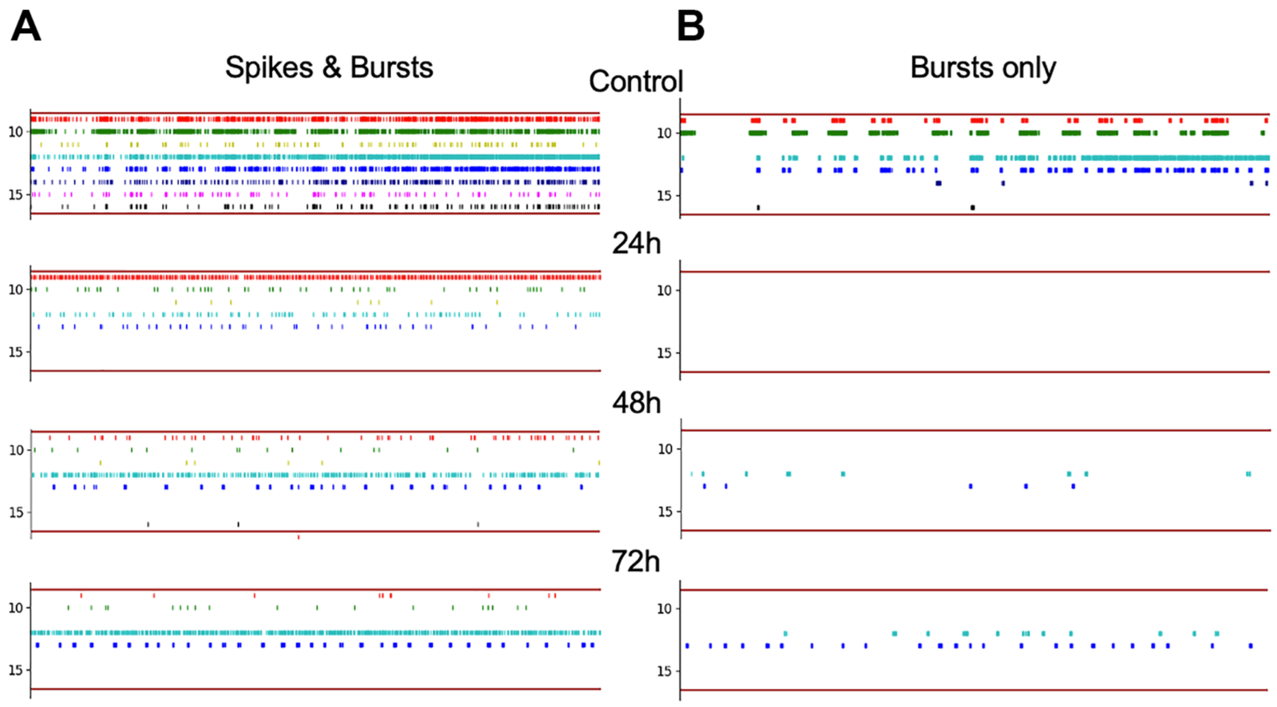

Supplementary Data S5: Time course analysis of the spontaneous activity from 20 months old (at start of the experiment) 3D neural tissues after the perfusion of trimethyltin chloride (TMT) 2.5 mM in a bottom Strip-MEA configuration. **A**) Raster plots of spike and burst activity in control and after 24 h, 48 h and 72 h. Each line represents an electrode (8 electrodes shown). Each bar of color represents a spike. **B**) Raster plot showing only burst activity with a complete absence of burst after 24 h although few bursts could be observed after 48 h and 72 h.

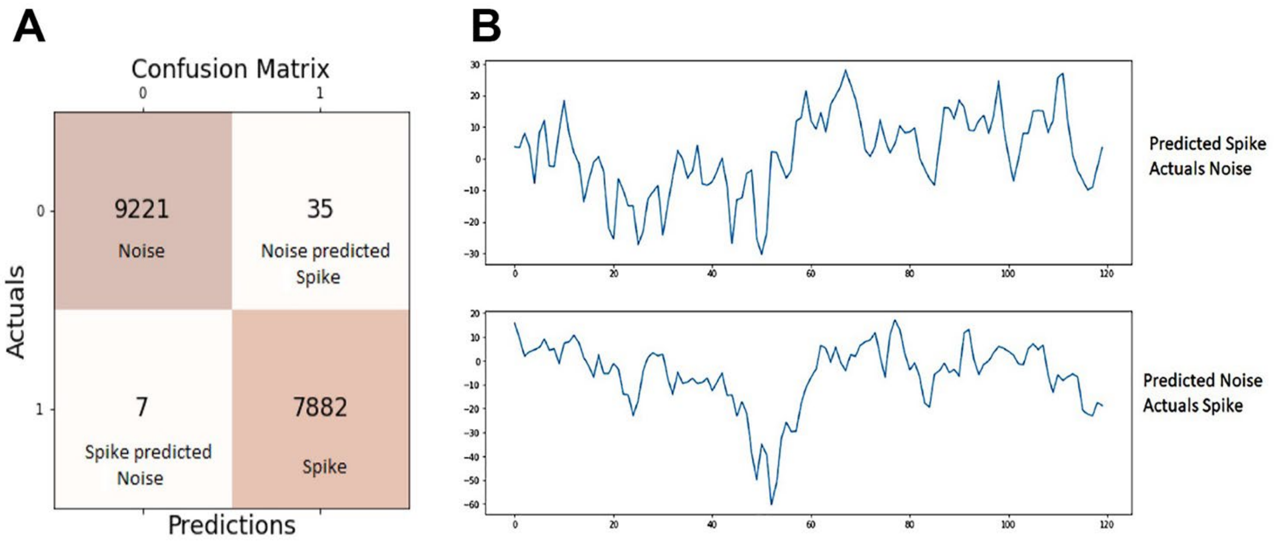

Supplementary Data S6: A) Confusion matrix Dense Classifier: As we can see with this example of Confusion Matrix, the model could categorize most of the signals. Out of 17,145 samples, the classifier made only 42 wrong predictions. This corresponds to 99.75% of correct predictions. The wrong predictions are partly due to bad labelling of the training dataset. However, results show that the model correctly classified most of the biological signals. **B**) Illustrations of wrong predictions of the Dense Classifier model. At top, the Dense Classifier model predicted and labelled the signal as a spike, but the signal corresponds to noise. At bottom, inversely, the Dense Classifier model predicted and labelled it as noise instead of a spike.

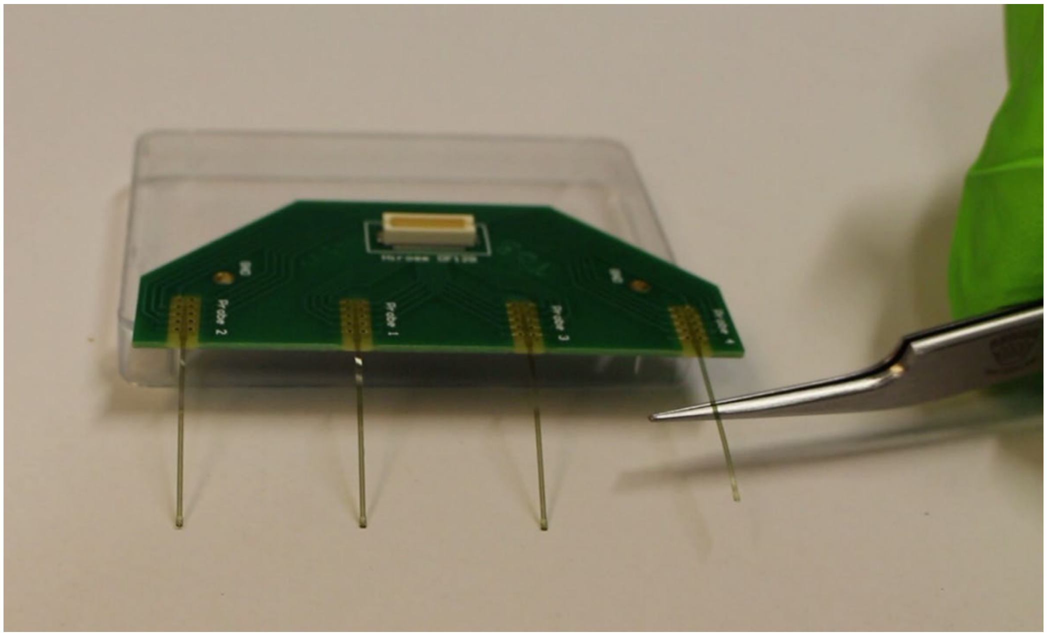

Supplementary Data S7: Short video showing a 4-Strip MEA biochip before its final assembly and illustrating that the Strip-MEA probes are flexible (see video file).
